# Mediator complex: the unsung hero of hematopoiesis

**DOI:** 10.3389/fcell.2026.1959663

**Published:** 2026-10-09

**Authors:** Nadezhda A. Kuznetsova, Elena V. Misnik, Ekaterina A. Varlamova, Alvina I. Khamidullina

**Affiliations:** Institute of Gene Biology Russian Academy of Sciences, Moscow, Russia

**Keywords:** CDK8/19, erythropoiesis, hematological disorders, hematopoiesis, leukemia, mediator complex

## Abstract

Hematopoiesis is a hierarchical process governed by complex transcriptional networks that dictate the self-renewal and lineage-specific differentiation of hematopoietic stem cells (HSCs). While not the sole central regulator, the Mediator complex plays a crucial role in this landscape as a highly conserved multi-protein assembly that transmits signals from enhancer-bound transcription factors to RNA polymerase II. Through its distinct structural modules, particularly the cyclin-dependent kinase module (CKM), Mediator acts as a crucial regulator of transcriptional initiation and elongation. Emerging evidence underscores the Mediator’s indispensable role in normal blood cell development and hematological pathologies. Genetic ablation of specific Mediator subunits severely compromises HSC stemness, impairs erythroid maturation, and disrupts embryonic circulatory development. Furthermore, Mediator dysregulation is intricately linked to the pathogenesis of malignant hematological disorders, especially leukemias. This review synthesizes current knowledge regarding the Mediator complex’s function in embryonic and adult hematopoiesis, with a primary focus on human and murine erythropoiesis. We critically examine the molecular mechanisms underlying hematological diseases driven by Mediator dysfunction and highlight the key complex’s components important for different blood pathologies. Finally, we outline future perspectives on targeting specific Mediator components, such as the CDK8/19 kinases, as promising therapeutic strategies for treating Mediator-dependent hematological diseases.

## Introduction

Hematopoiesis is a multistage process that ensures the continuous renewal of blood cells. In mammals, hemopoiesis is maintained by hematopoietic stem cells (HSCs), which are capable of self-renewal and differentiation into either myeloid or lymphoid lineages under the control of the HSC niche (Kandarakov et al., 2022; Cochrane et al., 2009). These lineage decisions occur independently but are regulated by overlapping transcription networks and signaling cascades (Mercer et al., 2011). Lymphopoiesis is the process of formation, maturation and differentiation of lymphocytes (T, B and NK cells) from stem cells. Myelopoiesis includes the stages of formation and development of all other formed elements of blood including granulocytes, macrophages, erythrocytes and thrombocytes (Cochrane et al., 2009). During hematopoiesis, HSCs and different progenitors undergo sequential maturation through multiple stages that proceed in a hierarchical manner (Schippel and Sharma, 2023). Thus, transcription regulation and reprogramming play an important role in the process of formation of blood cells.

The Mediator is a highly conserved multi-protein complex of about 1.4 MDa, consisting of four main modules: head, body, tail and cyclin-dependent kinase module (CKM) (Soutourina, 2018; Richter et al., 2022). The main function of the Mediator is to transmit the signal from enhancer-associated transcription factors to the components of the transcription machinery, stabilize the preinitiation complex and modulate the activity of RNA polymerase II (Soutourina, 2018; Richter et al., 2022; Friedson et al., 2025). CKM includes the cyclin-dependent paralog kinases CDK8/19, their partner cyclin C (CycC), as well as the proteins MED12/12L and MED13/13L. Depending on its association with the CKM, the Mediator acts as both a negative and a positive regulator of transcription initiation and facilitates transcription during the elongation stage as well.

In recent decades, there have appeared many studies shedding light on the key role of the Mediator in normal hematopoiesis and the development of pathological conditions. Knockout of several Mediator subunits has been shown to cause embryonic death due to defects in circulatory system development, alter the stem cell properties of HSCs and lead to a decrease in the number of erythroid precursors (Zhu et al., 2024; Lee et al., 2023; Aranda-Orgilles et al., 2016; Stumpf et al., 2010; Stumpf et al., 2006). The dysfunction of the Mediator is associated with the development of malignant hematological diseases (acute myeloid leukemia (AML), chronic lymphocytic leukemia (CLL), acute lymphoblastic leukemia (ALL), etc.) which makes the Mediator a promising therapeutic target.

Despite accumulating evidence on the important role of the Mediator complex components in hematopoiesis, research on this topic remains limited. This review summarizes and systematizes current data on the role of the Mediator from two important sides: normal embryonic and adult hematopoiesis and pathogenesis of hematological disorders outlining the perspectives for therapeutic targeting of Mediator complex components. Altogether, the main aim of the review is to draw attention to the broader study of the Mediator components for the improvement of approaches for treatment of hematological diseases.

## Stages of human and mouse hematopoiesis

Hematopoiesis is a multi-stage process necessary for the formation and differentiation of all blood cells in the body (Komic et al., 2025). Although blood cells are formed during both embryonic and postnatal development, these two processes differ significantly.

Embryonic hematopoiesis is divided into primitive hematopoiesis in the yolk sac (YS) and definitive hematopoiesis, originating in the aorta-gonad-mesonephros (AGM) region and later shifting to the fetal liver (Figure 1A). Primitive hematopoiesis produces large, nucleated erythrocytes, megakaryocytes, and macrophages, while definitive hematopoiesis generates all mature blood cell types and hematopoietic stem cells (HSCs). HSCs are a heterogeneous population of multipotent cells with variable self-renewal and differentiation potential, lacking a universal marker (Jurecic, 2019). Long-term repopulating HSCs (LT-HSCs, CD34^+^CD38^−^CD45RA-CD90^+^) sustain lifelong hematopoiesis and give rise to short-term HSCs and multipotent progenitors (MPPs), which have limited or no self-renewal (Wen et al., 2020; Skulimowska et al., 2022). MPPs differentiate into common myeloid and lymphoid progenitors, which further branch into lineage-restricted precursors, reflecting a gradual, transcription factor-driven hierarchy (Cheng et al., 2020; Skulimowska et al., 2022). However, this classical model is likely oversimplified, given intermediate progenitors with restricted potential and CD34^−^ HSCs directly differentiating into erythroid-megakaryocytic precursors (Schippel and Sharma, 2023).

**FIGURE 1:**
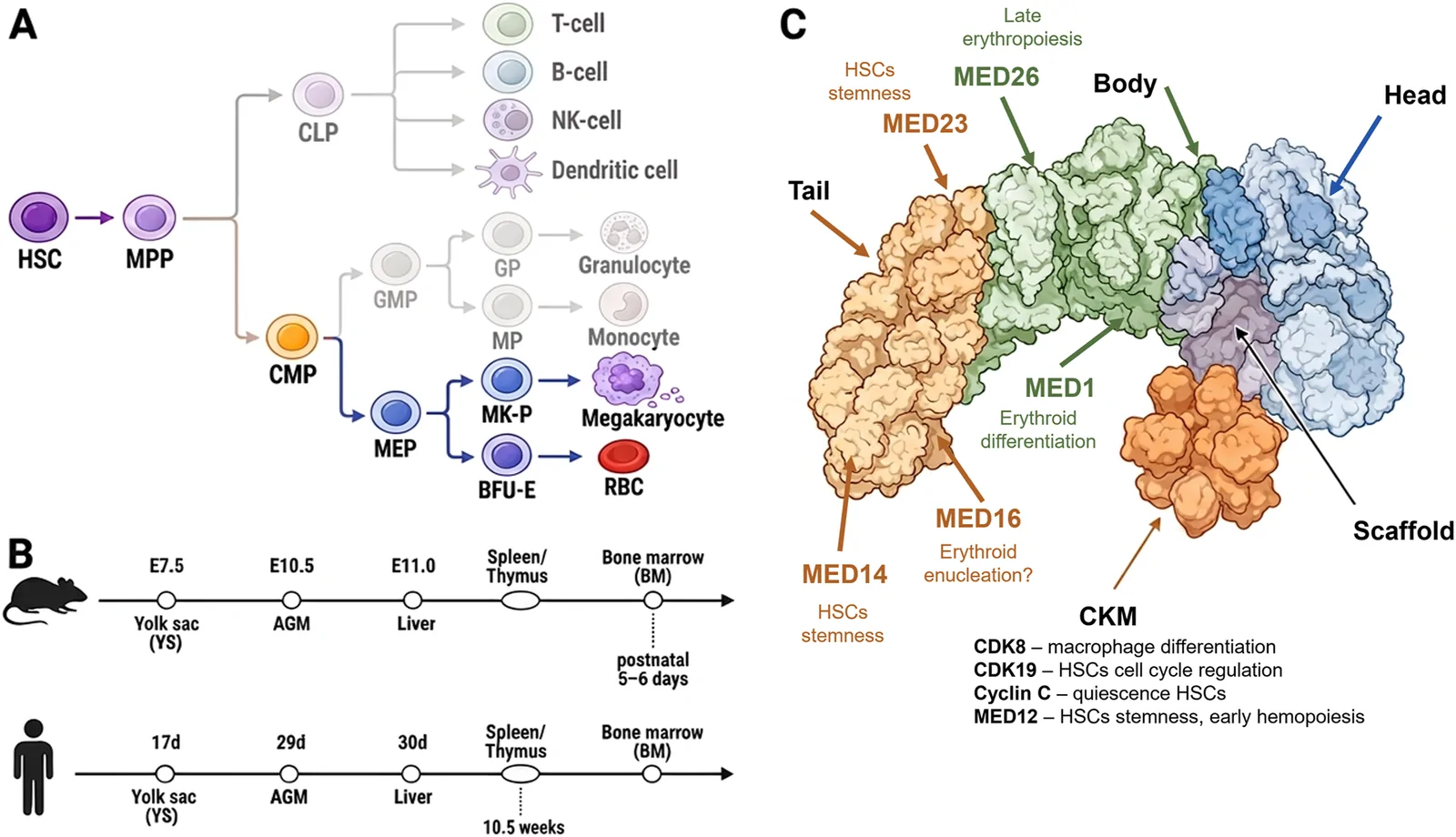
Overview of hematopoietic differentiation and the Mediator complex. **(A)** The hierarchical model of hematopoiesis. Hematopoietic stem cells (HSC) give rise to multipotent progenitors (MPP), which differentiate into the common lymphoid progenitor (CLP) and common myeloid progenitor (CMP) lineages. CLP generates T-cells, B-cells, NK-cells, and dendritic cells. CMP branches into the granulocyte-monocyte progenitor (GMP), producing granulocytes and monocytes via granulocytes progenitors (GP) and monocyte progenitors (MP) intermediates, and the megakaryocyte-erythroid progenitor (MEP), producing megakaryocytes and red blood cells (RBC) via megakaryocyte progenitors (MK-P) and burst-forming unit-erythroid (BFU-E) intermediates. **(B)** Hematopoiesis chronology during mouse and human development. Both species progress through sequential hematopoietic sites yolk sac (YS), aorta-gonad-mesonephros (AGM), fetal liver, spleen/thymus, and bone marrow (BM) with corresponding developmental timepoints indicated for mouse (embryonic days E7.5-E11.0, postnatal days 5–6) and human (gestational weeks 4.5–10.5). **(C)** Structure of the Mediator complex, comprising the tail, middle, and head modules, with the dissociable cyclin-dependent kinase module (CKM). Subunits implicated in hematopoietic regulation are highlighted. The figure was created using Noah AI.

In mice primitive hematopoiesis begins at E7.5 in YS blood islands, containing hemangioblasts derived from the primitive streak (Figure 1B) (Duan et al., 2019). Targeted disruption of genes involved in hemangioblast specification (*Lmo2*, *Scl*) leads to complete block of hematopoiesis and embryonic death (Palis, 2016). The YS is essential for forming the first erythroid cells, macrophages, and megakaryocytes (Ferkowicz et al., 2003). Clonogenic progenitors and precursors–CFU-GEMM (colony-forming unit-granulocyte, erythrocyte, macrophage, megakaryocyte), BFU-E (burst-forming unit-erythroid), CFU-E (colony-forming unit-erythroid), and CFU-GM (colony-forming unit–granulocyte, macrophage) – arise from CD34^+^ hemangioblasts within these islands shortly thereafter, by around E8.0-E8.5 (Stefanska et al., 2017). By E8.25, common erythromyeloid/lymphoid precursors emerge, capable of erythroid differentiation (Palis, 2016; Atkins et al., 2022). Unlike definitive erythropoiesis, primitive erythropoiesis generates erythroid cells that remain nucleated throughout maturation and co-express both embryonic (**ζ**, βH1, ɛy) and adult (ɑ1, ɑ2, β1, and β2) hemoglobins (reviewed in (McGrath and Palis, 2005)). Together with macrophages and megakaryocytes, these primitive erythroid cells constitute the three cell lineages generated during the primitive wave (Palis, 2016). Primitive hematopoiesis is HSC-independent; the first functional HSCs appear at E10.5-E11.5 from AGM endothelium (Palis, 2016; Lange et al., 2021; Dzierzak and Bigas, 2018). HSCs then migrate to the fetal liver (main hematopoietic organ from E11) (Chou and Lodish, 2010), then spleen/thymus (E13.5), and finally bone marrow (BM), formed E17.5-E18.5 (Cecchini et al., 1997; Gao et al., 2018). Long-term repopulating HSCs and niche establishment emerge only postnatally (days 4–5) (Tavian and Péault, 2005). BM remains active throughout life, though hematopoietic cell numbers decline with age (Prabhakar et al., 2009).

Human embryonic hematopoiesis goes through the same stages, but with some differences (Figure 1B). On the 16th day, thickening of the YS wall is observed. At 4.5 weeks of development, a number of clonogenic progenitors and precursors originating from CD34^+^ hemangioblasts in the blood islands appear. These populations are found between 35 and 50 of gestation and disappear entirely by day 60. Thus, the duration of hematopoiesis in the human YS is significantly shorter relative to that in mice (Tavian and Péault, 2005). As in mice, the first long-term repopulating HSCs in humans appear in the AGM region (E10.5–11.5 in mice, days 28–42 in human embryos) (Lewis et al., 2021). These cells are of mesodermal origin and are formed from hemogenic endothelial cells under the control of a number of transcription factors (TF) and cytokines. They undergo the epithelial-mesenchymal transition, enter the circulation and colonize the fetal liver. Concurrently, at week 5, the fetal liver is populated by HSCs and hematopoietic precursors migrating from the YS. Thus, the fetal liver contains both AGM HSCs providing active erythromyeloid hematopoiesis and YS HSCs, which are thought to maintain hematopoietic homeostasis during fetal development (Lewis et al., 2021). Some studies suggest that the liver is colonized twice: on day 23, CD34^−^erythromyeloid precursors appear in the hepatic sinusoids, and on day 30, CD34^+^ hematopoietic cells at later developmental stages are detected, which are able to establish hematopoiesis *in vitro* (Tavian and Péault, 2005).

BM is the primary site of postnatal hematopoiesis in humans in which hematopoiesis begins at the 11th week of gestation. CD15^+^ myeloid cells and CD235a+ erythrocytes are the first cells to be detected in the BM. As in mice, human blood cells likely populate the BM during the later stages of differentiation and develop independently of the osteoblastic niche which enables rapid hematopoiesis. Definitive HSC in the BM appears later (reviewed in (Tavian and Péault, 2005)).

Each stage of hematopoiesis is controlled by fine-tuned transcriptional programs, in which an “orchestra” of TFs sequentially guides stem and progenitor cells toward specific blood lineages (Wang et al., 2021). At the level of hemogenic endothelium and emerging HSCs, TFs such as SCL/TAL1 and RUNX1 are critical for establishing definitive hematopoiesis and initiating hematopoietic fate from multipotent precursors (Kwon et al., 2024; Gutiérrez et al., 2020; Porcher et al., 2017). GATA2 and FLI1, together with RUNX1 (Atkins et al., 2022), contribute to hematopoietic stem and progenitor cell survival and self-renewal, providing a transcriptional framework that keeps early progenitors in a stem-like state while allowing lineage priming (Wilson et al., 2010). As differentiation progresses toward myeloid lineages, PU.1 and CEBPA/B drive granulocytic and monocytic programs, shifting the balance away from lymphoid and stem cell signatures (Zhao et al., 2022; Theilgaard-Mönch et al., 2022). In contrast, erythroid and megakaryocytic commitment relies on GATA1 and KLF1, which activate globin genes and erythroid-specific transcriptional circuits (Borg et al., 2011; Woon Kim et al., 2011). LMO2 functions as a key scaffolding transcription factor in early hematopoiesis, forming multi-protein complexes with SCL/TAL1 and GATA factors to stabilize enhancer binding, promote hemogenic specification, and later support erythroid and megakaryocytic lineage commitment (Inoue et al., 2013). Together, these TFs form a hierarchical, context-dependent network that integrates signaling inputs with chromatin cofactors to ensure robust yet flexible control of hematopoietic differentiation.

## The Mediator complex and hematopoiesis regulation

The Mediator is a highly conserved multisubunit protein complex (26 subunits in mammals) linking transcription factors and enhancers to RNA polymerase II, thereby modulating transcription (Soutourina, 2018; Richter et al., 2022). It comprises head, body (MED1, MED26, etc.), tail (MED14, MED16, MED23, etc.), and a dissociable kinase module (CDK8/19, CycC, MED12/12L, MED13/13L), with head and body forming the minimal core needed for *in vitro* transcription (Verger et al., 2019; Malik and Roeder, 2023). Mediator acts as an intermediary between transcription factors and pre-initiation complex (PIC) components (TFIIB, TFIIH) and RNA polymerase II, promoting PIC assembly and chromatin remodeling to activate transcription (Soutourina, 2018; Luyties and Taatjes, 2022; Richter et al., 2022).

The functional kinase module of the Mediator complex contains CDK8/19 kinases, which activity requires CycC binding. Unlike canonical CDKs, T-loop phosphorylation is functionally replaced by MED12 binding (Klatt et al., 2020; Li et al., 2021). The CKM regulates gene expression both positively and negatively via the Mediator and transcription factor function at enhancers and promoters (Chen et al., 2023). Also, CKM acts as a cofactor and modifier of different transcription factors that coordinates the response to exogenous stimuli by reprogramming gene expression for optimal cell adaptation (reviewed in (Luyties and Taatjes, 2022)).

Investigations show the importance of different Mediator subunits and CKM in regulating hematopoietic differentiation. Most Mediator subunits (including head subunits, MED1, CycC, etc.) decrease in expression during terminal erythroid differentiation (Lee et al., 2023; Zhu et al., 2024).

MED1 is essential for erythropoiesis: its mutation causes embryonic lethality (E11.5) with reduced erythroid progenitors. MED1 can coactivate GATA1 for β-globin gene induction (Stumpf et al., 2010), though MED1 or GATA1 alone is sufficient to trigger differentiation independently (Mizuta et al., 2014; Pope and Bresnick, 2013). GATA1 also binds MED17 and MED14 (Stumpf et al., 2006). MED1 supports niche function, as *Med1* ^*−/−*^ stromal cells impair HSC proliferation and colony formation (Inoue et al., 2008; Sumitomo et al., 2010). Knockout of the *Med1* gene in the mouse YS decreases monocytic and myeloid differentiation of HSC due to suppression of vitamin D and retinoic acid receptor-dependent granulopoiesis (Urahama et al., 2005).

MED12 is the best-studied subunit in context of regulation of hematopoiesis by Mediator: its deletion causes T lymphocytes to lose stemness (Freitas et al., 2022), impairs colony formation in CD34^+^ cells (Aranda-Orgilles et al., 2016), and reduces HSCs numbers and their colony-forming ability in mouse embryonic liver, without affecting on the functioning mouse embryonic fibroblasts or embryonic stem cells. MED12 interacts with p300/CBP to drive expression of *Runx1*, *Gata2*, and *Fli1* in HSPCs, while deletion of other kinase-module components genes (*Med13*, *Ccnc*, *Cdk8*) does not affect HSC survival and colony-forming ability, indicating MED12 has kinase-module-independent functions (Aranda-Orgilles et al., 2016).

Although CKM components are not required for HSC survival, they are required for its fate determination. CDK19 regulates HSC cell cycle via p21/p53 signaling: its loss decreases p21 expression without changes in p53 levels, suggesting modulation of p53 activity, slowing HSC division and reducing HSCs numbers (Zhang Z. et al., 2022). Also, CDK8/19 inhibition causes cardiac and circulatory defects in zebrafish (Uehara et al., 2020), postnatal Cdk8 deletion in mice has no major hematopoietic effect (Menzl et al., 2019) emphasizing species variability in the functions of the CKM. Specific postnatal *Cdk8/19* double knockout only in hematopoietic cells increases splenic macrophages frequency in mice and alters their cytokine profile and phagocytosis without severe hematopoietic disease (Kolodziejczyk et al., 2025). *CCNC* knockout in human CD34^+^ HSCs increases quiescence (G0) and engraftment potential without blocking differentiation (Miyata et al., 2010).

Like MED1, MED26 regulates the early stages of erythropoiesis by affecting the survival and proliferation of hematopoietic stem cells. MED26 affects early HSC survival and proliferation and later drives transcriptional pausing of RNA polymerase II (via NELF, DSIF, PAF) to accelerate erythroid differentiation (Lee et al., 2023; Zhu et al., 2024). Although it is known that CDK8 also plays an important role in maintaining the promoter-proximal pausing (Steinparzer et al., 2019; Varlamova et al., 2025), the corresponding molecular mechanism of CDK8 regulation of erythropoiesis has not yet been described.

MED23 plays a role in maintaining the stemness of HSCs and suppressing differentiation. Its knockout in mice biases toward myelopoiesis and reduces lymphoid output by repressing *Cebpa* and sustaining *Scf*, *Sca1*, and *Cd34* expression (Chen X. et al., 2018).

Preliminary data indicates that loss of MED16 in murine models enhances erythroid and myeloid colony formation and accelerates erythroid enucleation. However, data on the role of MED16 in the regulation of erythropoiesis require more detailed consideration and study of the molecular mechanisms and are extremely promising for study (Xie et al., 2023).

Taken together, these data suggest that the Mediator regulates hematopoiesis through at least three distinct mechanisms rather than a single unified pathway. First, CKM subunit MED12 can at least partially act as scaffolding components whose hematopoietic function is separable from the CKM catalytic activity. Other catalytic subunits themselves (CDK8, CDK19, CycC) show context-dependent effects. Individual knockouts produce selective, often cell-type- or tissue-restricted phenotypes, and these effects vary substantially between embryonic and postnatal contexts and across species. Third, non-CKM Mediator subunits (MED1, MED16, MED23, MED26) appear to function via core Mediator-dependent and CKM-independent mechanisms. These subunits either directly coactivate lineage-determining transcription factors or regulate Pol II pausing, and their loss results in reproducible defects that are confined to particular stages of differentiation.

## The Mediator complex subunits in hematological disorders

Life-threatening hematopoiesis pathologies are divided into deficiencies of different blood cell types (e.g., anemia is deficiency of healthy erythrocytes) and malignancies. A significant portion of hematopoiesis disorders are non-severe forms of anemias, while among the rarer but more dangerous diseases there are many leukemias. They have various genetic and non-genetic causes, and transcriptional regulation can play a role through various mechanisms (reviewed in (Khamidullina et al., 2021)).

Myelodysplastic syndromes (MDS) are clonal HSC disorders causing ineffective hematopoiesis, marrow dysplasia, and cytopenias, with risk of progression to acute myeloid leukemia (AML). The annual incidence of MDS increases more than sixfold among people over 65 years of age (Sekeres and Taylor, 2022). MDS therapy is aimed at eliminating dominant cytopenias and is designed to reduce the need for blood transfusions. Depending on the severity of the disease, erythropoiesis stimulating agents and hypomethylating drugs such as azacitidine and decitabine, as well as HSCs transplantation, are used for therapy (Sekeres and Taylor, 2022). The disadvantages of this treatment are side effects, including anemia (Liu and Karlsson, 2024).

Leukemias are heterogeneous hematological malignancies driven by uncontrolled proliferation of transformed hematopoietic precursors in bone marrow (BM) and blood, progressively disrupting normal HSC self-renewal and differentiation. As the disease progresses, leukemic cells progressively displace normal hematopoietic cells, disrupting the self-renewal and differentiation of HSCs and progenitors. According to the current classification, leukemias are divided into four major subtypes differ in origin, molecular pathogenesis, and treatment: acute lymphoblastic leukemia (ALL), acute myeloid leukemia (AML), chronic lymphocytic leukemia (CLL), and chronic myeloid leukemia (CML) (Huang et al., 2022). Leukemia accounted for 2.4% of new cancer cases and 3.1% of cancer deaths globally in 2022 (Bray et al., 2024).

In recent years, Mediator complex alterations affecting MED1, MED12, and the CKM increasingly emerge as either consequences or drivers of hematologic malignancies, disrupting transcription factor interactions in leukemogenesis.

MED1 is critical in B-cell ALL (B-ALL) driven by the E2A-PBX1 fusion oncoprotein (t (1; 19)): MED1 interacts with E2A-PBX1 recruiting Mediator to genes supporting leukemic survival and proliferation. *MED1* knockout selectively suppresses expression of E2A-PBX1 target genes and inhibits E2A-PBX1-positive leukemia growth (Lee et al., 2021). MED1 is necessary as well for optimal transcription initiated by PML-RARα–another fusion protein resulting due to chromosomal translocation t (15; 17) (q22; q12) and causing the development of acute promyelocytic leukemia, a highly aggressive subtype AML. MED1 also coactivates PML-RARα-driven transcription in acute promyelocytic leukemia, dependent on all trans retinoic acid treatment, highlighting therapeutic relevance (Fukuoka et al., 2019).

MED12 dysregulation occurs in AML and CLL. In AML subtypes, DNA hypermethylation aberrantly activates MED12 transcription (Chavan et al., 2025). In CLL, *MED12* mutations correlate with adverse prognosis, activate NOTCH signaling (Wu et al., 2017), associate with unmutated immunoglobulin heavy-chain variable genes and *ZAP70* kinase expression (Kämpjärvi et al., 2015), while disrupting Mediator-CKM interaction and kinase activity (Turunen et al., 2014; Kämpjärvi et al., 2015).

CDK8 is frequently deregulated in hematological malignancies (Xu and Ji, 2011), restraining superenhancer-associated genes in AML (Pelish et al., 2015) and driving CDK8-dependent STAT1 Ser727 phosphorylation that promotes AML proliferation (Nitulescu et al., 2017). CDK8 knockdown prolongs B-ALL latency, disrupts mTOR signaling, and sensitizes cells to pathway inhibitors (Menzl et al., 2019). In SF3B1-mutant MDS, splicing factor mutations impair CDK8 mRNA splicing, reducing CDK8 and skewing HSC differentiation toward erythroid and myeloid lineages (Bonner et al., 2025). The study of CDK8 is interesting not only as an antitumor target, but also may be considered as a target in anti-anemia therapy. CDK8/19 inhibition restores MYC-driven ribosomal gene expression in Diamond-Blackfan Anemia models (Chen J. et al., 2018).

CycC is required for B-ALL leukemogenesis despite being dispensable for normal hematopoiesis. Its ablation abolishes leukemogenic potential via p53 signaling, while CycC high levels are associated with poor B-ALL outcomes and relapse risk (Trifinopoulos et al., 2025). CycC loss also accelerates T-cell ALL development, as mutant CycC/CDK8/19 complexes fail to phosphorylate Notch1 intracellular domain (Li et al., 2014). Altogether, these illustrate context-dependent strategies of Mediator subunit inactivation or *vice versa* activation in leukemia.

## Inhibitors of CDK8/19 as therapeutics of hematological disorders

The identification of Mediator complex dysregulation in hematological malignancies has driven development of selective CDK8/19 inhibitors (CDK8/19i) targeting the CKM (Table 1). Small-molecule inhibitors of the Mediator kinases CDK8/19 are being actively developed by different groups for therapeutic applications in various malignancies, especially in blood cancers (Philip et al., 2018). Their development was driven by their effectiveness in hematological disorders therapy coupled with low side effects, which is important given the elderly population and comorbidities of patients.

**TABLE 1:** Most prospective CDK8/19 inhibitors for hematological disorders and malignancies treatment.

Compound name	Development stage	Treatment regime	Indication	Key references
Cortistatin A	Preclinical (*in vitro*)	Monotherapy	AML	Pelish et al. (2015)
​	Preclinical (*in vitro*, *in vivo*)	Combination with ruxolitinib	Myeloproliferative neoplasms	Nitulescu et al. (2017)
Romaciclib (SEL120, RVU120)	Phase 1b clinical trial (NCT04021368, United States)	Monotherapy	AML; myeloproliferative neoplasms	Marańda et al. (2023)
​	Phase 2 clinical trial (NCT06243458, France)	Monotherapy	Lower-risk MDS	​
​	Phase 2 clinical trial (NCT06397313, Italy)	Monotherapy or combination with ruxolitinib	Intermediate- and high-risk primary or secondary myelofibrosis	​
​	Phase 2 clinical trial (NCT06268574, Italy)	Monotherapy	AML or higher-risk MDS	Papayannidis et al. (2024)
​	Phase 2 clinical trial (NCT06191263, France)	Monotherapy or combination with venetoclax	AML	Montesinos et al. (2025)
​	Phase 2 clinical trial (NCT06987058, Poland, Spain)	Monotherapy	AML or higher-risk MDS	​
​	Preclinical (*in vitro*, *in vivo*)	Monotherapy	AML	Rzymski et al. (2017)
​	Preclinical (*in vitro*, *in vivo*)	Combination with cytarabine	AML	Borthakur et al. (2019)
​	Preclinical (*in vitro*, *in vivo*)	Combination with venetoclax	MDS	Pakulska et al. (2021)
​	Preclinical (*in vitro*, *in vivo*)	Combination with pelabresib	AML	Statkiewicz et al. (2026)
Senexin C	Preclinical (*in vitro*, *in vivo*)	Monotherapy	AML	Zhang L. et al. (2022)

Cortistatin A was among the first compounds demonstrating therapeutic potential of CDK8/19i (Pelish et al., 2015), but its complex synthesis prompted development of simpler analogs (Hatcher et al., 2018). The Senexin series followed, with Senexin C showing improved metabolic stability and effective suppression of AML cell growth with favorable tolerability (Zhang L. et al., 2022). While AML is highly sensitive to CDK8/19i, approximately half of subtypes remain resistant. Several studies currently suggest various markers of AML sensitivity to CDK8/19i, such as transcription factor STAT5 or DNA methyltransferases (Rzymski et al., 2017; Rzymski et al., 2021). The sensitivity of AML cells to CDK8/19i may also depend on the metabolic characteristics of leukemic cells (Varlamova et al., 2026).

Romaciclib (SEL120, RVU120) is the most clinically advanced CDK8/19i, showing potent antileukemic activity via STAT5 phosphorylation inhibition (Rzymski et al., 2017; Abboud et al., 2022; Marańda et al., 2023; Statkiewicz et al., 2026). Beyond cytotoxicity, it induces differentiation: downregulating stemness genes and activating erythroid differentiation programs and hemoglobin metabolism in MDS-patients-derived CD34^+^ cells (Pakulska et al., 2021). In preclinical models, romaciclib reduced both the viability and abundance of the CD34^+^CD38^−^ cell population enriched for leukemic stem cells while simultaneously promoting their myeloid differentiation (Mazan et al., 2023). Early clinical trials in relapsed and refractory AML and high-risk MDS showed acceptable safety with responses including reduced blast burden and complete remission (Abboud et al., 2021; Abboud et al., 2022; Marańda et al., 2023).

CDK8/19i show promise in combination regimens: sensitizing cells to JAK1/2 inhibitor ruxolitinib in myeloproliferative neoplasms (Nitulescu et al., 2017), antimetabolite cytarabine and BCL2 inhibitor venetoxlax in AML and MDS (Borthakur et al., 2019; Pakulska et al., 2021), BCR-ABL1 inhibitors in CML (Khamidullina et al., 2025), acting synergistically with BET inhibitor pelabresib (Statkiewicz et al., 2026), and enhancing CAR-T (Freitas et al., 2022). Novel CDK8/19i romaciclib has entered clinical trials for solid tumors and leukemias (Table 1).

In Diamond-Blackfan Anemia, screening identified Senexin A/B, CCT251545, and romaciclib as stimulators of erythroid proliferation and differentiation in murine models and patient-derived CD34^+^ cells (RPS19, RPS26, RPL35A mutations), with pharmacological inhibition showing minimal effects on normal hematopoietic cells under the tested conditions (Chen J. et al., 2018). This apparent tolerability contrasts with genetic deletion studies, which reveal several physiological roles for CDK8/19 in normal hematopoiesis (see above). The discrepancy likely reflects differences between the two approaches. Pharmacological inhibition blocks only catalytic activity while leaving the protein’s non-catalytic (e.g., scaffolding) functions intact, whereas genetic deletion causes complete permanent loss of the protein and all its functions. It should also be noted that pharmacological inhibition is typically partial and limited by both dose and duration. Consequently, the effects of more prolonged or complete kinase inhibition on hematopoietic cells remain to be fully defined.

CDK8/19i also modulate immune responses, enhancing NK cell cytotoxicity (via increasing the expression of perforin/granzyme B) and potentiating anti-PD-1 therapy efficacy in preclinical tumor models (Hofmann et al., 2020).

Overall, preclinical studies support CDK8/19 inhibition as a promising therapeutic strategy in AML, although substantial heterogeneity in sensitivity may limit its efficacy. Importantly, clinical evidence remains preliminary, underscoring the need for further studies to define the determinants of response and the therapeutic potential of CDK8/19 inhibitors in AML.

## Conclusion

Subunits of the Mediator complex, such as MED1, MED23, and MED26, play key roles in both embryonic and postnatal hematopoiesis: their loss dramatically impairs the stemness of HSCs, erythroid cell maturation, and embryonic viability. The CKM is a significant regulator of HSCs cell cycle and their fate. Presented in this review data indicate both that CKM has unique functions independent of the core Mediator complex and that CKM subunits may have their own distinct molecular targets in hematopoiesis. Moreover, the Mediator kinases CDK8/19 and CycC are often implicated in the development of hematopoietic malignancies. Pharmacological inhibition of CDK8/19 activity allows for selective targeting of malignant cells with minimal disruption of normal hematopoiesis. Thus, the presence of a kinase target accessible to targeted therapy in the Mediator complex makes it a promising candidate for further study in the context of antitumor therapy for hematopoietic malignancies.
